# Optimal dynamic pricing for public transportation considering consumer social learning

**DOI:** 10.1371/journal.pone.0296263

**Published:** 2024-01-31

**Authors:** Yihua Zhang, Zhan Zhao

**Affiliations:** 1 Liuzhou Railway Vocational Technical College, Liuzhou, Guangxi, China; 2 Shandong University Stomatology Hospital, Jinan, Shandong, China; Universidad de Sevilla Facultad de Ciencias Económicas y Empresariales: Universidad de Sevilla Facultad de Ciencias Economicas y Empresariales, SPAIN

## Abstract

Effective public transportation pricing strategies are critical to reducing traffic congestion and meeting consumer demand for sustainable urban development. In this study, we construct a dynamic game pricing model and a social learning network model for consumers of three modes of public transportation including metro, bus, and pa-transit. In the model, the metro, bus, and pa-transit operators maximize their profits through dynamic pricing optimization, and consumers maximize their utility by adjusting their travel habits through social learning in the social network. The reinforcement learning algorithm is applied to simulate the model, and the results show that: (1) as consumers’ perceived sensitivity to different modes of travel increases, the market share and price of each mode of travel adjust accordingly. (2) When taking into account consumers’ social learning behavior, the market share of metros remains high, while the market shares of buses and pa-transit are relatively low. (3) As consumers become more sensitive to their perception of each travel mode, operators invest more resources in improving service quality to gain market share, which in turn affects the price of each travel mode. Our results provide decision support for optimal pricing of urban public transportation.

## 1. Introduction

With accelerated urbanization, urban traffic issues have become one of the common concerns faced by major cities [1]. As a core component of urban transportation, public transportation systems not only relieve traffic congestion and reduce traffic pollution, but also improve the sustainability and quality of life in cities [2, 3]. In addition, rational public transportation contributes significantly to the development of urban economies by facilitating the flow of human capital [4]. Public transportation such as metros, buses, and pa-transit (such as taxis, carpooling, etc.) play an important role in modern urban life, providing residents with convenient, fast, and affordable travel options [5, 6]. However, the balance between public transportation pricing and market demand is an issue that requires critical deliberation. Excessively high fares can lead to a decline in public transportation ridership, which reduces public transportation revenue and thus affects the sustainability of public transportation. Too low fares, on the other hand, may lead to losses in public transportation operations and fail to protect the normal operation of public transportation.

In fact, different operating companies tend to optimize their services by constantly adjusting their prices and other strategies in order to maximize their own profits. Among the many factors that influence public transportation pricing, competition with peers is one of the key factors [7]. To compete for market share, there is inevitably head-to-head competition between various modes of transportation. Therefore, it is important to accurately evaluate the price/performance gap with competitors, as well as the advantages and disadvantages of competing in the industry when pricing. To make reasonable considerations and decisions on these factors, it is necessary to develop a pricing strategy suitable for urban public transportation and improve the sustainability and competitiveness of public transportation.

Consumers’ perception of different modes of travel is increasingly becoming one of the most important factors to consider in competition. With the development of society and the continuous upgrading of consumers, consumers’ requirements for travel modes are gradually improving [8]. In addition to focusing on traditional factors such as price and convenience, modern consumers also focus on aspects such as travel comfort, environmental protection, and safety [9, 10]. Therefore, public transportation operators need to understand consumers’ needs in-depth and continuously innovate and improve their travel products and services to meet consumers’ changing needs and requirements. In addition, the role of social learning in travelers’ perception of public transportation cannot be ignored. Social learning is the process of learning knowledge and skills from the experiences and behaviors of others through communication, observation, and imitation [11]. Because public transportation services are oriented to the whole society, public attitudes and needs towards public transportation can influence and learn from each other [12]. Through social learning, the public can better understand the quality and value of public transportation services and increase their awareness and support for public transportation. Mutual learning interactions among travelers are usually ignored, which becomes inappropriate because intergroup relations widely influence travelers’ choice of public transportation modes through social contact.

Driven by this motivation, we develop a model to study the pricing strategies of transit operators and the travel choice behavior of consumers. Our model considers the competitive relationships among the three main modes of public transportation, including metro, bus, and pa-transit, as well as consumers’ perceived sensitivity and social learning behavior for each mode of travel. Our main contributions can be summarized as follows. Firstly, this study constructs a model that integrates the competitive relationships among metros, buses, and pa-transit, as well as consumers’ perceived sensitivity and social learning behavior for each mode of travel. The model provides a theoretical framework for understanding the dynamics of the public transportation market and helps public transportation operators and regulators to develop effective strategies and policies. Secondly, we examine the impact of changes in consumers’ perceived sensitivity to different modes of travel on the market share and prices of these modes. This analysis provides valuable insights for public transportation operators to adjust their pricing strategies and improve service quality to remain competitive in the market. Lastly, by incorporating consumer social learning into the simulation analysis, we are able to more accurately reflect consumer behavior and their perceived sensitivity to different modes of travel.

The rest of our study is organized as follows. Section 2 reviews the relevant literature. The model setup and algorithmic process are presented in Section 3. In Section 4, we perform a simulation analysis and also a sensitivity analysis. We conclude the paper in Section 5.

## 2. Literature review

### 2.1 Public transportation pricing

Starting with the seminal paper by Mohring [13], a large number of studies analyzing the optimal pricing and service delivery of public transportation modes have been arising. Jara-Diaz and Gschwender [14] constructed a microeconomic model to optimize bus pricing and operations. Wang, An [15] proposed a multi-modal, resilient, and balanced transportation model for strategic transportation management that explicitly includes pricing schemes to optimize urban transportation. Some of the studies have developed a series of numerical models to assess whether current public transportation fares and service levels are above or below the socially optimal levels. In some cases, it has been found that optimal fares are likely to be lower than current fares. De Borger and Wouters [16] and Proost and Van Dender [17] found that the best fares in Belgium and the city of Brussels were lower than the actual fares, respectively. As a result, optimal pricing for public transportation has attracted extensive attention in the literature. Karlaftis and McCarthy [18] argued that transit attributes are heterogeneous and have different production technologies and discussed cost-oriented pricing strategies. Buttazzo, Pratelli [19] sought an optimal pricing policy for the use of a public transportation network in a specific densely populated area and discussed the existence and some qualitative properties of the optimal pricing policy. Simic, Gokasar [20] defined four alternative public transport pricing systems, namely, flat fares, distance-based fares, regional fares, and rent-based fares, and prioritized these alternatives.

In recent years, a number of scholars have integrated the frontier tools of modern economics into the transportation field level and achieved some inspiring theoretical results. explored in depth the use of game theory in the direction of transportation and concluded that game theory is a powerful tool for analyzing transportation systems. Tamannaei, Zarei [21] proposed a mixed-integer linear programming model to study the competitive cargo transportation pricing problem based on the Stackelberg leader-follower competition noncooperative game-theoretic approach. Wu, Zhang [22] analyzed the pricing game between railroad companies and bus companies under free competition from the perspective of profit maximization. Gong, Ren [23] used a game framework to analyze optimal wholesale rail public transport prices. Gao, Mazalov [24] use game theory to study the equilibrium traffic flow problem for urban public transport passengers, including buses, trolleybuses, trams, metros, pa-transit, and bicycles.

In addition, some researchers have begun to focus on passengers’ willingness to pay as the most critical influencing factor in public transport pricing. Pepper, Spitz [25] estimated passengers’ willingness to pay for congestion reduction based on empirical tests. Molin and Timmermans [26] noted that travel information for passenger public transportation is highly price sensitive. Increasingly, transportation studies are beginning to use stated preference data to gain accurate knowledge about traveler preferences. Beirao and Cabral [27] conducted a qualitative study on personal characteristics, lifestyle, type of activity, and level of service of transportation modes to derive the main factors that influence the mode of travel of passengers by public transport and minibusses. Shiftan, Outwater [28] analyzed the time-sensitivity of travelers to investigate the causal relationship between latent and explicit variables of residents’ intention to travel. Eriksson and Forward [9] found that travelers’ attitudes and perceived behaviors are important determinants of transportation mode. Neven, Braekers [29] studied the impact of customized bus services on elderly and disabled people based on simulation methods and analyzed the factors that passengers consider when choosing customized bus trips. Zhang, Wang [30] analyzed the factors affecting the choice of customized bus trips, and the study showed that fares, bus lanes, and travel time have a significant impact on the choice of customized bus service mode.

### 2.2 Consumer social learning

Bandura [11] first introduced the concept of social learning and defined it as the act of learning important information by observing the behavior of other members. Consumer social learning is the process by which consumers learn and acquire knowledge through social interaction and information sharing when purchasing products or services [31]. Consumer social learning is an important area in consumer behavior research that can help companies better understand consumer needs and behaviors [32]. Factors influencing consumer social learning include social networks, personal trust, information reliability, and cognitive ability. Through social networks, consumers can share their shopping experiences and advice with others and obtain a wider range of information and feedback [33, 34]. Consumer trust and information reliability are also important factors influencing social learning, as consumers tend to trust sources that have a reliable reputation and word of mouth [35]. In addition, consumers’ cognitive ability is a key factor in social learning, as they need to be able to understand and apply what they have learned to make informed purchasing decisions [36].

Consumer social learning is of great importance to companies. By understanding consumer social learning, companies can better understand consumers’ needs and behaviors [37]. Based on this, companies can take appropriate measures to improve the quality and satisfaction of their products or services, thereby enhancing consumer loyalty and word-of-mouth [38]. At the same time, companies can also use consumer social learning to develop more effective marketing strategies and pricing strategies, thereby increasing market share and profitability [39]. In addition, by effectively communicating and interacting with consumers, companies can also promote consumer social learning and provide consumers with a better shopping experience.

Along this line, the literature has examined the impact of consumer social learning behavior on firms’ pricing strategies. Papanastasiou and Savva [40] found that in the absence of social learning, firms always tend to choose to decrease price plans, while in cases where social learning has a significant impact, pre-announced pricing policies usually do not benefit the firm. Crapis, Ifrach [41] analyzed social learning mechanisms and their effect on sellers’ pricing decisions impact. Numerical experiments show that pricing policies that take social learning into account can significantly increase revenue relative to policies that do not take social learning into account. Jing [42] investigated the impact of social learning (SL) on dynamic pricing and consumer adoption of durable goods in a two-period monopoly and showed that firms are likely to benefit from informational advertising or investment to foster more social learning. Qiu and Whinston [43] examined the optimal pricing strategies of monopolistic firms in considering consumer social learning behavior. The results show that offering introductory discounts on social networks is not always an effective way to promote purchases. Xiao, Zhang [44] constructed a two-period duopoly model of an innovative product and investigated the impact of consumer social learning on firms’ pricing strategies and profits.

However, there are also gaps in existing related research that need to be filled. Firstly, dynamic pricing of public transport can help optimize resource utilization, improve the financial sustainability of the public transport system, and enhance the passenger experience to better respond to changing transport demand. Although existing studies have used game theory approaches to analyze the pricing strategy of public transportation, there is no literature that uses dynamic game approaches to analyze the dynamic pricing problem. Secondly, understanding consumers’ perceived sensitivity to each mode of travel can help public transport operators optimize their pricing strategies, but the existing literature lacks a coherent framework for an in-depth analysis. Finally, it is clear from the existing literature that consumers’ social learning behaviors have a significant impact on firms’ pricing strategies. Nevertheless, this has been overlooked in the existing literature when modeling public transport pricing.

## 3. The model and algorithm process

### 3.1 Pricing came analysis for different modes of transportation

In modern cities, the demand for transportation is growing rapidly, and people have an urgent need for efficient, comfortable, and convenient transportation methods. Among them, metros, buses, and pa-transit are the three main public transportation modes [45, 46]. Let the set *A* = {*Metro*, *Bus*, *Pa*−*transit*} represent these three modes of transportation, each with their unique advantages, such as the high speed of metros, the economy of buses, and the flexibility of pa-transit. For a specific route, we assume that there are N consumers who need to travel, with the set represented as *C* = {1,2,⋯,*N*}. Each time a consumer travels, they face the choice of different transportation modes. In this paper, the probability of consumer *i* choosing transportation mode *j*∈*A* is denoted as *ρ*_*ij*_, as shown below [47]:

$$\rho_{ij}=\frac{e^{u_{ij}}}{\sum_{j}e^{u_{ij}}}$$ (1)

Where, *u*_*ij*_ represents the utility of consumer *i* choosing transportation mode *j*∈*A* for their trip.

Typically, the factors affecting consumer travel decisions are quite intricate and complex. We divide them into two major categories: the cost of travel for passengers (including time and monetary costs) and the passengers’ perceptions (such as safety, convenience, comfort, and accessibility). To uniformly represent these factors in the model, we convert them into value units and use a utility function to describe their combined effects, as shown below [48]:

$$u_{ij}=\alpha_{ij}T_{ij}+\beta_{ij}P_{j}+\gamma_{ij}F_{ij}$$ (2)

Where, *T*_*ij*_, *P*_*j*_, *F*_*ij*_ represent the time cost, monetary cost, and other perceptions for consumer *i* when choosing transportation mode *j*∈*A*, respectively; *α*_*ij*_, *β*_*ij*_, *γ*_*ij*_ represent the sensitivity of consumer *i* to time cost, monetary cost, and other perceptions when choosing transportation mode *j*∈*A*, respectively.

The time cost *T*_*ij*_ of consumer *i* choosing transportation mode *j*∈*A* for their trip consists of in-vehicle time and out-of-vehicle time, expressed as [49]:

$$T_{ij}=s_{i}^{w}t_{ij}^{w}+s_{i}^{c}t_{ij}^{c}$$ (3)

Where, $t_{ij}^{w}$ represents the out-of-vehicle time for consumer *i* when choosing transportation mode *j*∈*A*, which is the time it takes for consumer i to reach the boarding point and wait for the vehicle. $t_{ij}^{c}$ represents the in-vehicle time for consumer *i* when choosing transportation mode *j*∈*A*, which is the time spent by the consumer traveling with transportation mode *j*. $s_{i}^{w}$ represents the sensitivity of consumer *i* to the out-of-vehicle time. The larger $s_{i}^{w}$ is, the more consumer *i* is concerned about the time it takes to reach the boarding point and wait for the vehicle. $s_{i}^{c}$ represents consumer *i*’s perception of the value of in-vehicle time.

The out-of-vehicle time $t_{ij}^{w}$ is related to the distance and speed of the consumer reaching the boarding point, as well as the frequency of the transportation mode, expressed as [50, 51]:

$$t_{ij}^{w}=\frac{d_{ij}}{v_{w}}+\theta f_{j}$$ (4)

In the model, *d*_*ij*_ represents the distance between consumer *i* and the boarding point of transportation mode *j*, *v*_*w*_ represents the walking speed of the consumer, and *f*_*j*_ represents the average departure interval of transportation mode *j*. Additionally, for the sake of simplicity in this study, we assume that the arrival pattern of urban passenger flow follows a uniform distribution, and within a certain operating time range, the departure interval of urban rail transit is fixed. Therefore, the waiting time for consumers at the boarding point is the expected departure interval of transportation mode *j*, that is, *θ* = 0.5.

The in-vehicle time $t_{ij}^{c}$ is related to the travel distance and speed of consumer *i* when using transportation mode *j*, expressed as [50, 51]:

$$t_{ij}^{c}=\frac{L_{ij}}{v_{cj}}$$ (5)

In the model, *L*_*ij*_ represents the travel distance of consumer *i* when using transportation mode *j*, and *v*_*cj*_ represents the average speed of transportation mode *j*.

The monetary cost factors affecting consumer travel are determined by the operating companies of the metro, bus, and pa-transit transportation modes. As can be seen from Eq 2, the price of transportation mode *j*∈*A* is represented as *P*_*j*_, so the revenue of the operating company for transportation mode *j* can be expressed as [49–51]:

$$\pi_{j}=P_{j}\sum_{i=1}^{N}x_{ij}$$ (6)

Where, *x*_*ij*_∈{0,1}. If consumer *i* chooses transportation mode *j*, then *x*_*ij*_ = 1; otherwise, it is equal to 0. The operating companies of the three transportation modes compete for customers and maintain their market share by optimizing their prices.

### 3.2 Social learning model

In the decision-making process of consumers’ travel, social networks play a crucial role. Social networks not only include daily interactions between consumers but also interactions in online social media (such as Weibo, WeChat, etc.). Through social networks, consumers can observe others’ travel modes and evaluations of transportation options like metros, buses, and pa-transit, thereby adjusting their travel preferences.

In general, social learning is an essential component of consumer decision-making, and this learning mainly relies on social networks. The social networks here refer to complex networks established based on interpersonal relationships, where each person is a node, and social relations are the edges connecting these nodes. Thus, in this study, the social network for consumers to engage in social learning is defined as [52, 53]:

$$EN=\left[\begin{array}{ccc} e_{11} & \cdots & e_{1N}\\ \vdots & \ddots & \vdots \\ e_{N1} & \cdots & e_{NN} \end{array}\right]$$ (7)

Where *e*_*ij*_∈{0,1}, *e*_*ij*_ = 1 means there is a social relationship between consumers *i* and *j*, and vice versa there is no social relationship. This paper is based on the assumption of the Six Degrees of Separation theory, which posits that the relationship network among consumers follows a specific structure where the connection between any two consumers does not exceed six intermediaries. Building upon this theory, the paper constructs a small-world network as the social network model used in the simulation study. In this small-world network, the connections among consumers exhibit a highly interconnected pattern, allowing for swift connections to be established even between any two nodes in the network through a small number of intermediary nodes. The establishment of such a network structure will aid us in gaining a deeper understanding of information dissemination, social interactions, and decision-making processes among consumers, thereby providing more precise and powerful tools and methods for market research and social network analysis.

Consumer *i*’s sensitivity to the time cost, monetary cost, and safety, convenience, comfort, and accessibility aspects of transportation option *j*∈*A* is influenced by other consumers, as shown below [34, 54]:

$$\alpha_{ij}≔\omega \alpha_{ij}+(1-\omega )\frac{1}{N_{i}}\sum_{k\in E_{i}}\alpha_{ij}$$ (8)

$$\beta_{ij}≔\omega \beta_{ij}+(1-\omega )\frac{1}{N_{i}}\sum_{k\in E_{i}}\beta_{ij}$$ (9)

$$\gamma_{ij}≔\omega \gamma_{ij}+(1-\omega )\frac{1}{N_{i}}\sum_{k\in E_{i}}\gamma_{ij}$$ (10)

where *ω* represents the degree to which consumers are influenced by the opinions of other consumers, *E*_*i*_ = {*k*|*e*_*ij*_ = 1} represents the set of all consumers having a social relationship with consumer *i*, and *N*_*i*_ represents the number of elements in set *E*_*i*_.

### 3.3 Algorithm process

DDPG is a reinforcement learning algorithm that uses deep neural networks to learn how to formulate the optimal strategy in a continuous action space. The core of this algorithm is the utilization of an experience buffer to enhance the efficiency and stability of learning. In each training step, DDPG stores the agent’s experiences (including state, action, reward, and the next state) in the experience buffer. Then, it randomly samples small batches of data from the experience buffer to train the network model. This approach reduces the correlation between data, making the training process more stable.

In this study, we use the DDPG algorithm model to build multiple agents, simulating the pricing process of metro, bus, and pa-transit transportation operators, respectively. In each game round, the three operators use historical data from the experience buffer to train neural networks and make pricing decisions, which are influenced by market demand and the pricing strategies of competitors. Consumers evaluate the overall utility of various transportation options based on their current perception of different transportation modes in terms of time cost, monetary cost, and other aspects (such as comfort, safety, etc.), and make their travel decisions accordingly.

**Fig 1:**
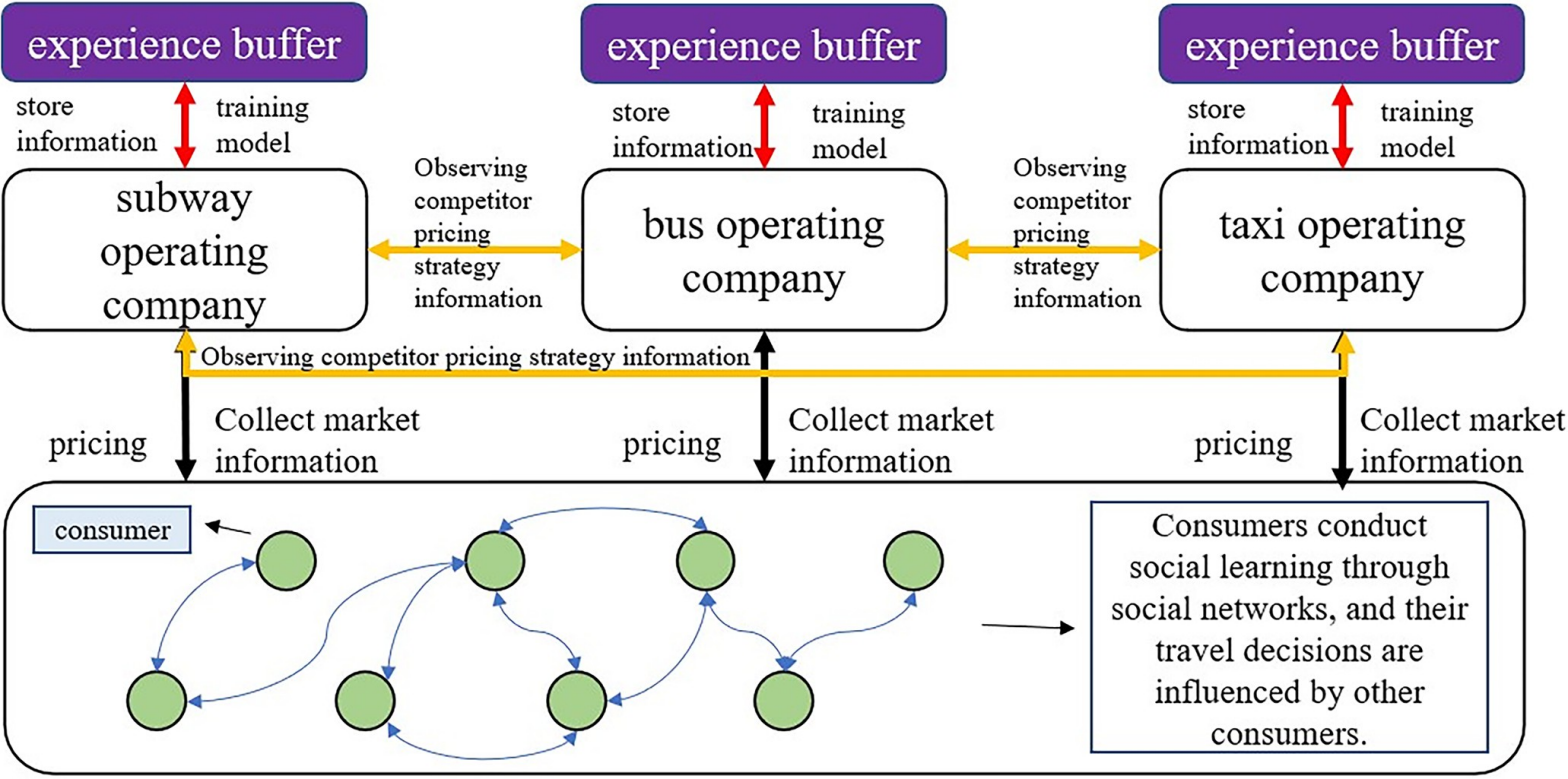
Algorithm process.

In this process, each operator continually adjusts their pricing strategy to maximize profits by observing consumers’ travel choices and competitors’ pricing strategies. When faced with different price and service options, consumers learn from the experiences of other consumers through social networks and weigh various factors to make appropriate travel decisions (as shown in Fig 1). As the game rounds progress, a dynamic equilibrium gradually forms between operators and consumers, reflecting the interaction between market competition and consumer demand.

## 4. Simulation analysis

### 4.1 Model parameter settings

The initial parameter settings for this study’s model were obtained through the distribution of questionnaires, with a total of 728 distributed and 695 collected. The content of the questionnaire is detailed in the Appendix. Through this consumer public transportation survey, we gained a deep understanding of consumers’ preferences in different travel modes, their satisfaction and evaluation of public transportation, and the factors they consider when choosing transportation options, such as distance, cost, comfort, congestion, safety, convenience, and accessibility. In addition, the questionnaire also covers information on consumers’ monthly income and whether they would refer to others’ travel methods. According to the results of the received questionnaire survey, this article calculates the mean and standard deviation of each question and uses the 95% confidence interval as the range of initial parameter values. For example, for question 2 in the survey questionnaire ("How sensitive are you to the time cost of the following means of transportation?"), the statistical mean for option A is 1.01, with a standard deviation of 0.538. Therefore, the 95% confidence interval is [0.97, 1.05]. This sets the range of consumer sensitivity to the time cost of metro travel to [0.97, 1.05]. Similarly, for question 7 in the survey questionnaire ("What are your average travel distances by metro, bus and pa-transit?"), the statistical mean for option B is 8.094, with a standard deviation of 89.9027. This results in a 95% confidence interval of [1.41, 14.78]. After rounding, the range of values for consumer travel distance for bus travel is set to [1, 15]. The settings for other parameters follow the same approach as described above. In summary, the initial parameter settings for this article are summarized in Table 1.

**Table 1:** Parameters.

*j* = *Metro*			
*α*_*ij*_	0.97–1.05	*f*_*j*_	3-5min
*β*_*ij*_	0.81–0.96	*L*_*ij*_	5–40 kilometers
*γ*_*ij*_	1.42–1.48	*v*_*cj*_	Between 30 km and 50 km per hour
*F*_*ij*_	6.3	*d*_*ij*_	Between 500m and 1000m
*j* = *Bus*			
*α*_*ij*_	0.75–0.83	*f*_*j*_	5-10min
*β*_*ij*_	1.08–1.16	*L*_*ij*_	1–15 kilometers
*γ*_*ij*_	1.44–1.52	*v*_*cj*_	Between 20 km and 30 km per hour
*F*_*ij*_	5.1	*d*_*ij*_	Between 300m and 500m
*j* = *Pa*−*transit*			
*α*_*ij*_	0.72–0.84	*f*_*j*_	0 min
*β*_*ij*_	1.01–1.13	*L*_*ij*_	2–30 kilometers
*γ*_*ij*_	1.32–1.41	*v*_*cj*_	Between 30 km and 50 km per hour
*F*_*ij*_	7.5	*d*_*ij*_	0 m
Others			
$s_{i}^{w}$	0.87–1.53	*v*_*w*_	5 kilometers per hour
$s_{i}^{c}$	2.17–2.94		

### 4.2 Baseline model simulation analysis

**Fig 2:**
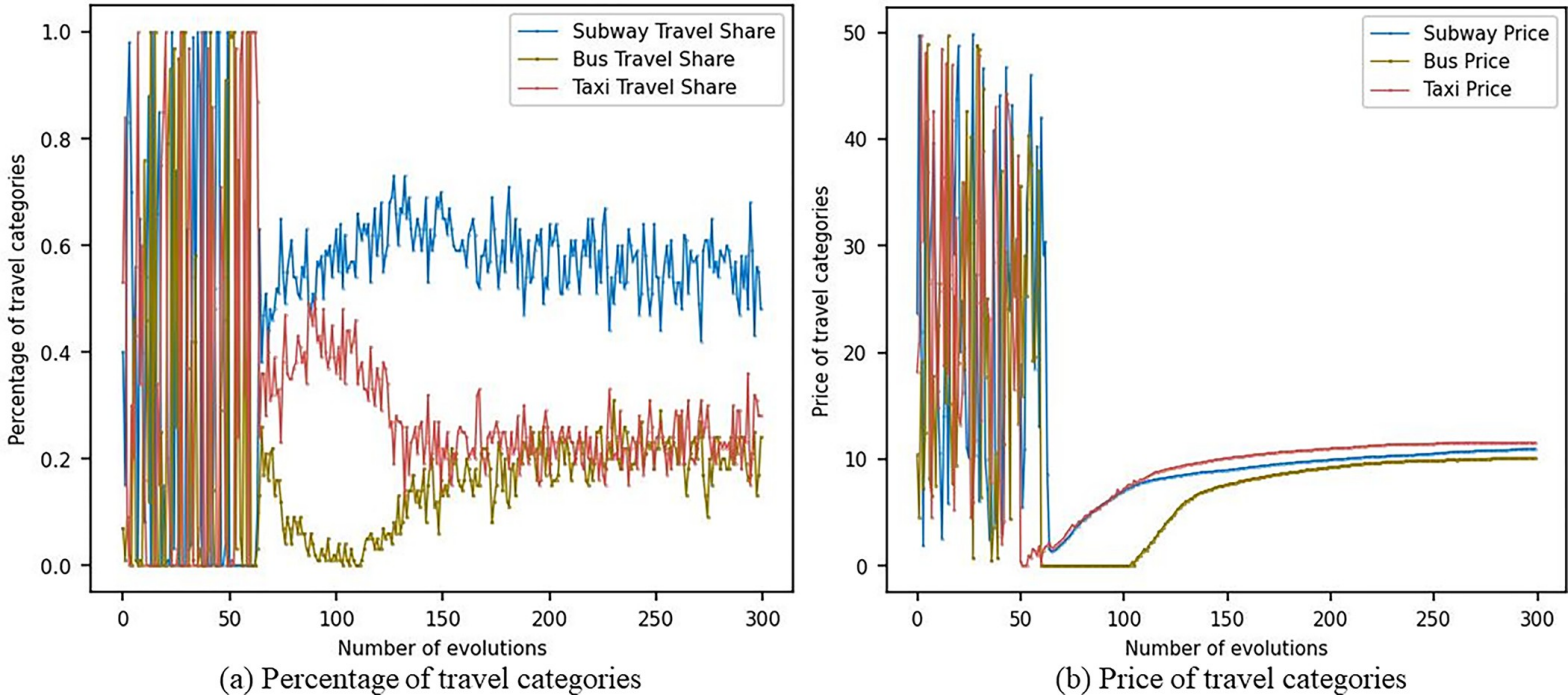
Baseline model simulation results.

Based on the above parameter settings, this study conducted 300 simulation rounds, demonstrating the pricing changes and market share changes of metro, bus, and pa-transit operators, as shown in the Fig 2 below.

As can be seen from Fig 2(A), with the increase in the number of simulations, the number of people choosing to travel by metro gradually increases, and its market share stabilizes at around 0.6. The market shares of both bus and pa-transit travel modes are roughly equal, both at around 0.2, with pa-transit slightly higher. From Fig 2B), it is evident that pa-transit fares are the highest, followed by metro fares, and bus fares are the lowest. This is mainly because the time and monetary costs of taking the metro are relatively low, especially during peak hours and congested sections, where the metro attracts a large number of passengers with its high speed and punctuality. Additionally, although metro fares are higher than bus fares, they are still lower than pa-transit fares, making them competitive. Moreover, the metro performs relatively well in terms of safety, convenience, comfort, and accessibility, causing more and more people to choose it as their mode of transportation. The performance of buses and pa-transit in these areas is relatively weaker, which may result in their lower market shares. Furthermore, metro, bus, and pa-transit operators may strive for market share by continually adjusting their prices. In this process, the metro operator may have found an optimal balance between time and monetary costs, stabilizing its market share at around 0.6. In contrast, the pricing strategies of buses and pa-transit may not be as successful, resulting in their lower market shares.

### 4.3 Sensitivity analysis

To further understand and explain the mechanisms of changes in consumer travel decisions and public transportation operators’ pricing decisions, this study conducted sensitivity analyses from multiple perspectives.

#### 4.3.1 Sensitivity analysis of metro travel perception without social learning

**Fig 3:**
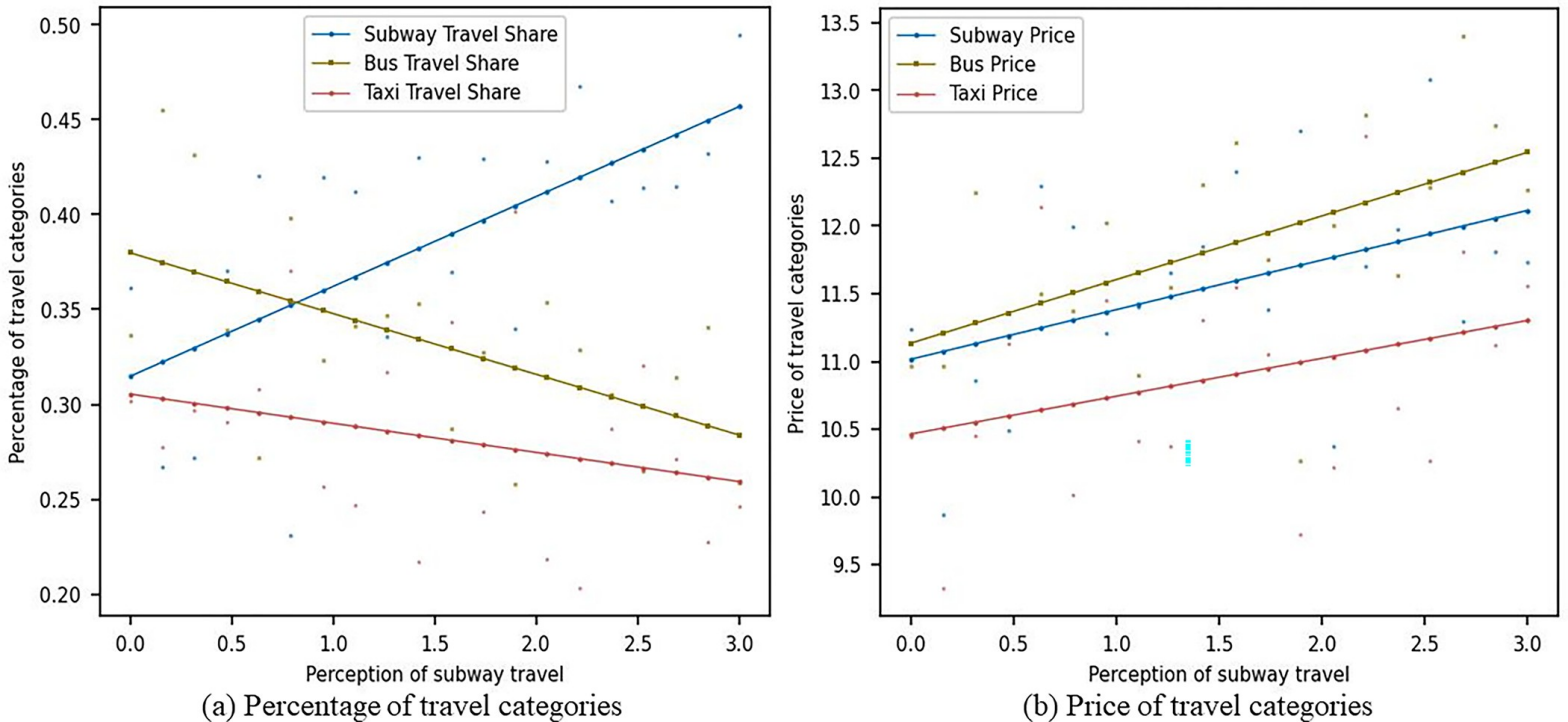
Sensitivity analysis of metro travel perception without social learning.

Fig 3(A) and 3(B) shows the impact of changes in consumers’ sensitivity to metro travel perception on the market share and prices of metro, bus, and pa-transit travel modes in the scenario without considering consumer social learning. As seen in Fig 3(A), as consumers’ sensitivity to metro travel perception increases, the market share of metro travel gradually rises from around 0.3 to around 0.45. The market share of bus travel gradually declines from around 0.37 to around 0.27, while the market share of pa-transit travel gradually falls from 0.3 to around 0.25. In Fig 3(B), as consumers’ sensitivity to metro travel perception increases, the prices of all three travel modes show a gradually increasing trend. An increase in consumers’ sensitivity to metro travel perception means that they pay more attention to the convenience, comfort, and other aspects of metro travel. In this case, the market share of metro travel gradually increases, indicating that the metro better meet consumers’ needs. Accordingly, the market shares of bus and pa-transit travel gradually decline, suggesting a weakened position of these two travel modes in consumer demand. Moreover, as consumers’ sensitivity to metro travel perception increases, the prices of all three travel modes show a gradually increasing trend. This may be due to increased investments by companies in the pursuit of market share to improve service quality and meet consumer demand. In this competitive environment, companies need to constantly adjust their pricing strategies to maintain competitiveness. As consumers’ sensitivity to metro travel perception increases, service quality and consumer satisfaction become key competitive factors for all travel modes. metro, bus, and pa-transit operators need to pay attention to changes in consumer demand, improve service quality, increase consumer satisfaction, and maintain market share.

#### 4.3.2 Sensitivity analysis of metro travel perception with social learning

**Fig 4:**
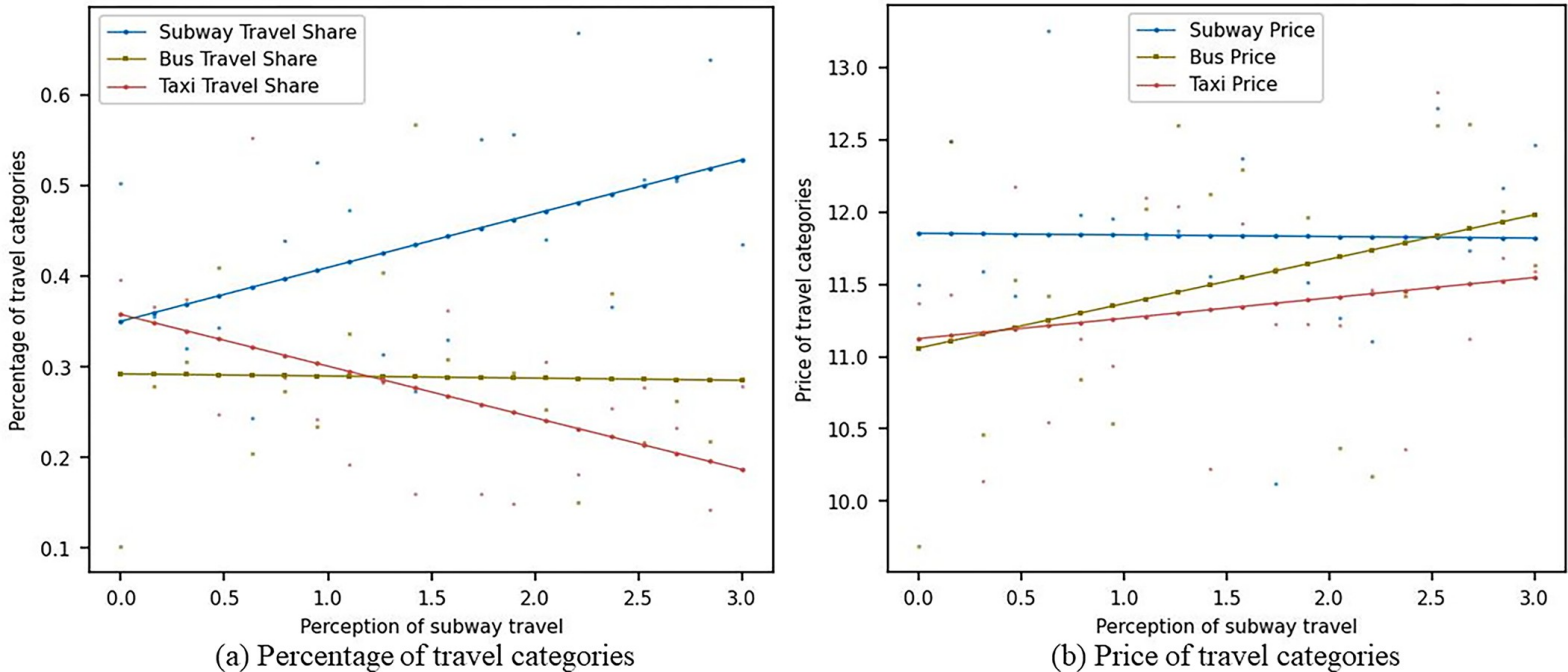
Sensitivity analysis of metro travel perception with social learning.

Fig 4(A) and 4(B) shows the impact of changes in consumers’ sensitivity to metro travel perception on the market share and prices of metro, bus, and pa-transit travel modes in the scenario considering consumer social learning. As seen in Fig 4(A), as consumers’ sensitivity to metro travel perception increases, the market share of metro travel gradually rises from around 0.35 to around 0.55. The market share of bus travel slightly declines and stabilizes around 0.3, while the market share of pa-transit travel gradually falls from 0.35 to around 0.15. In Fig 4(B), as consumers’ sensitivity to metro travel perception increases, metro prices gradually decrease from 12 to around 11.5, bus prices gradually increase from 11 to around 12, and pa-transit prices gradually increase from 11.2 to around 11.5. In comparison, consumer social learning increases the market share of metro travel and lowers the prices of all three travel modes. This means that consumer social learning can help disseminate information about metro advantages, thereby increasing metro market share and, to some extent, lowering the prices of all three travel modes. For various transportation companies, this implies the need to pay attention to consumer social learning behavior and demand changes, adjust pricing strategies, and improve service quality to maintain competitiveness in fierce market competition. At the same time, governments and regulatory authorities also need to closely monitor changes in consumer behavior to develop corresponding policies and regulatory measures.

#### 4.3.3 Sensitivity analysis of bus travel perception without social learning

**Fig 5:**
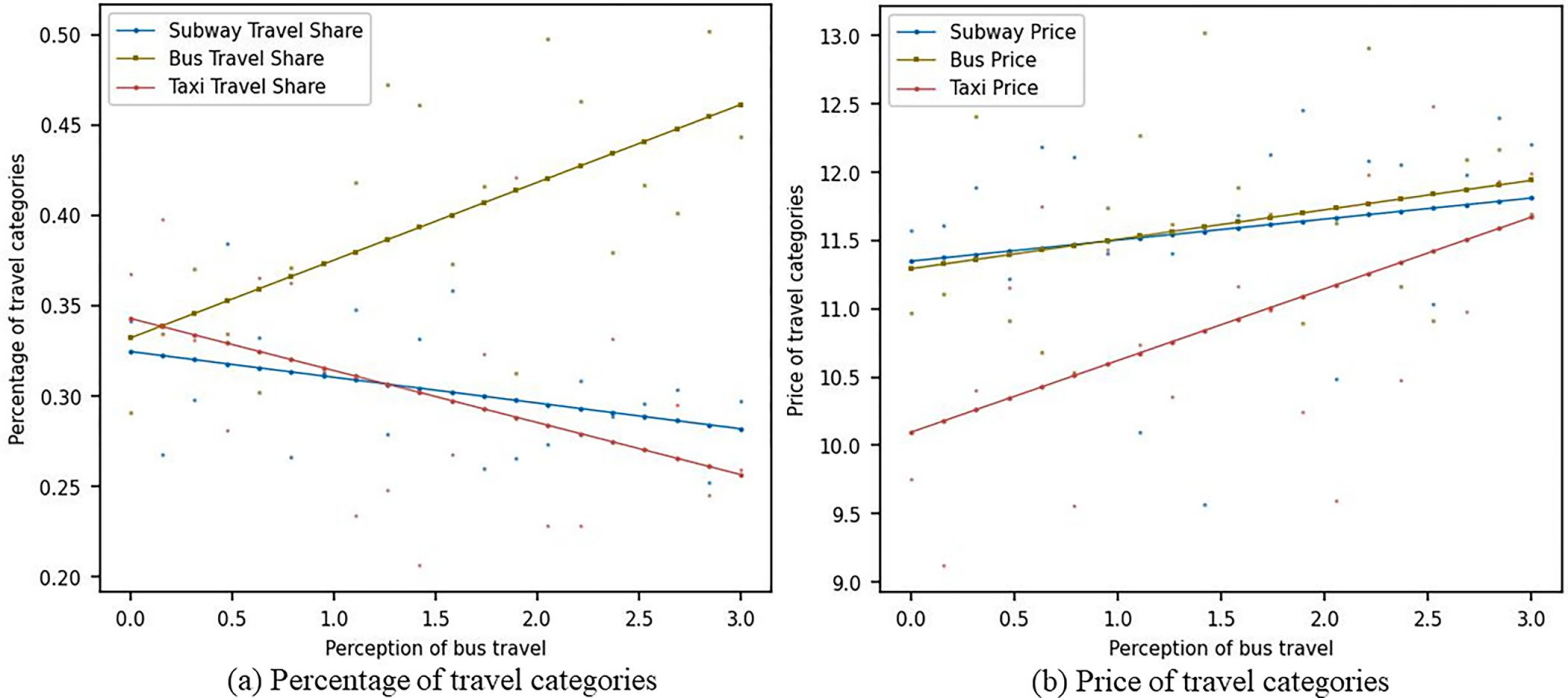
Sensitivity analysis of bus travel perception without social learning.

Fig 5(A) and 5(B) shows the impact of changes in consumers’ sensitivity to bus travel perception on the market shares and prices of metro, bus, and pa-transit travel modes under the scenario without considering social learning. In Fig 5(A), as the sensitivity of consumers to bus travel perception increases, the market share of metro travel gradually decreases from around 0.33 to 0.25, the market share of bus travel gradually increases from around 0.33 to 0.47, and the market share of pa-transit travel decreases from 0.33 to around 0.30. In Fig 5(B), as the sensitivity of consumers to bus travel perception increases, the prices of all three travel modes gradually increase. The prices of buses and metros are very close, both rising from 11.5 to around 12, while the price of pa-transit increases from 10 to 12. As consumers become more sensitive to bus travel perception, they may be more inclined to try bus travel. This behavior change may be influenced by various factors, such as personal experience and recommendations from friends. Moreover, metro, bus, and pa-transit travel modes may face different degrees of market segmentation. For example, the metro may dominate the long-distance travel market, buses may dominate the short-distance travel market, and pa-transit may dominate the travel market with higher flexibility requirements. In this scenario, metro, bus, and pa-transit companies may need to invest more resources in technological innovation to improve service quality and attract consumers. For example, bus companies can invest in intelligent and low-carbon technologies to improve operational efficiency and environmental performance.

#### 4.3.4 Sensitivity analysis of bus travel perception with social learning

**Fig 6:**
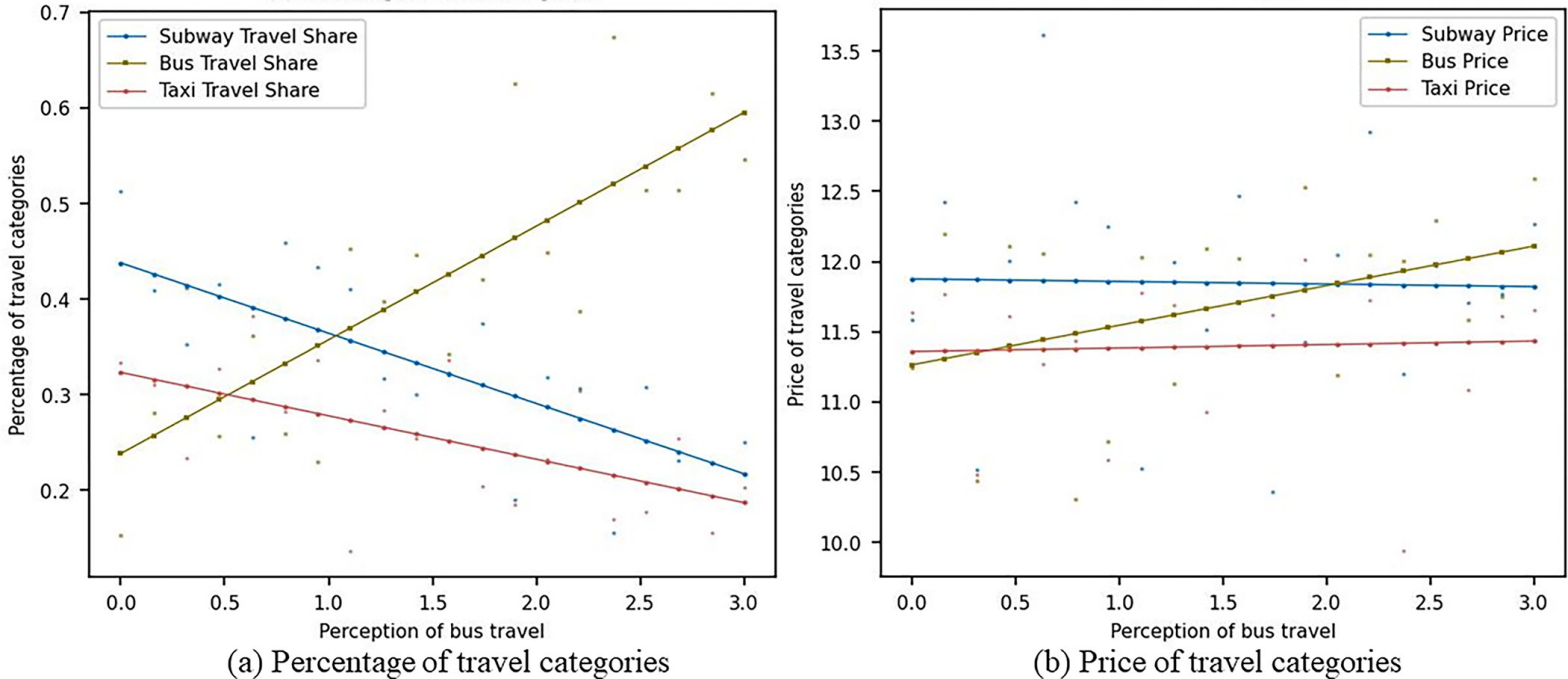
Sensitivity analysis of bus travel perception with social learning.

Fig 6(A) and 6(B) shows the impact of changes in consumers’ sensitivity to bus travel perception on the market shares and prices of metro, bus, and pa-transit travel modes under the scenario considering social learning. In Fig 6(A), as the sensitivity of consumers to bus travel perception increases, the market share of metro travel gradually decreases from around 0.45 to 0.2, the market share of bus travel gradually increases from around 0.25 to 0.6, and the market share of pa-transit travel decreases from 0.45 to around 0.25. In Fig 6(B), as the sensitivity of consumers to bus travel perception increases, the prices of metro and pa-transit remain relatively stable, while the price of bus travel gradually increases from 11 to around 12. This suggests that under social learning conditions, although consumers prefer metro travel, as the sensitivity to bus travel perception increases, consumers will gradually adopt buses as their primary mode of travel. Interactions among consumers can promote information dissemination, enabling them to understand and accept the advantages of bus travel. As sensitivity to bus travel perception increases, consumers may more easily learn about the convenience and comfort of bus travel from social networks, word of mouth, or other channels, and thus switch to bus travel. In the process of social learning, individuals may be influenced by group behavior, especially when facing insufficient information or high uncertainty. When consumers see more and more people choosing bus travel and expressing high satisfaction, they may be influenced by group behavior and become more inclined to choose buses as their mode of travel.

#### 4.3.5 Sensitivity analysis of pa-transit travel perception without social learning

**Fig 7:**
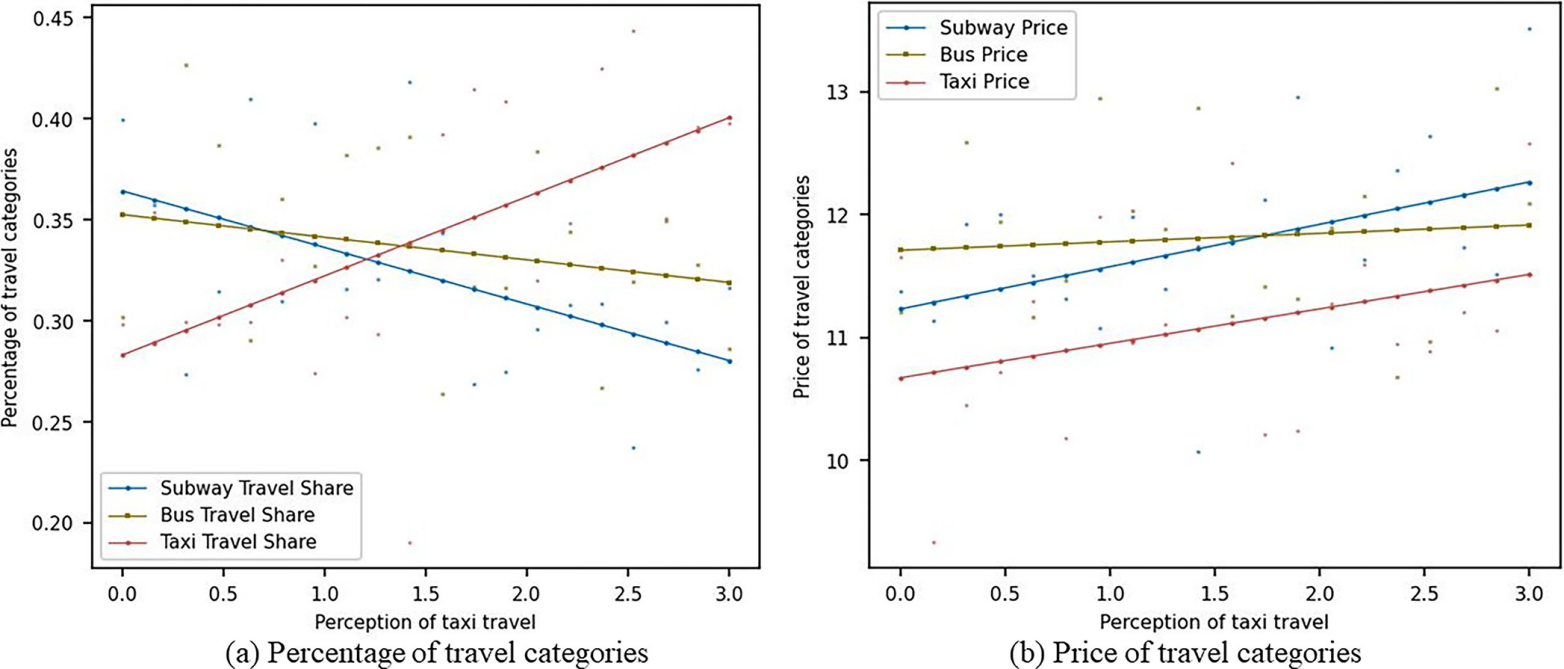
Sensitivity analysis of pa-transit travel perception without social learning.

Fig 7(A) and 7(B) shows the impact of changes in consumers’ sensitivity to pa-transit travel perception on the market shares and prices of metro, bus, and pa-transit travel modes under the scenario without considering social learning. In Fig 7(A), as the sensitivity of consumers to pa-transit travel perception increases, the market share of metro travel gradually decreases from around 0.37 to 0.32, the market share of bus travel gradually decreases from around 0.35 to 0.3, and the market share of pa-transit travel gradually increases from 0.27 to around 0.4. In Fig 7(B), as the sensitivity of consumers to pa-transit travel perception increases, the prices of the metro, bus, and pa-transit all gradually increase. When consumers become more sensitive to pa-transit travel perception, they may pay more attention to the advantages of pa-transit, such as personalized service, private space, and flexibility. This change in value perception may lead consumers to prefer pa-transit travel. As consumers become more sensitive to pa-transit travel perception, their demand for metro and bus travel may relatively decrease. This may be due to the different services provided by pa-transit compared to metros and buses, and consumers may believe that pa-transit can better meet their needs. Additionally, as consumers become more sensitive to pa-transit travel perception, competition among metros, buses, and pa-transit may become more intense. In order to attract consumers, various travel modes may improve service quality or adopt other strategies, thereby causing prices to gradually rise.

#### 4.3.6 Sensitivity analysis of pa-transit travel perception with social learning

**Fig 8:**
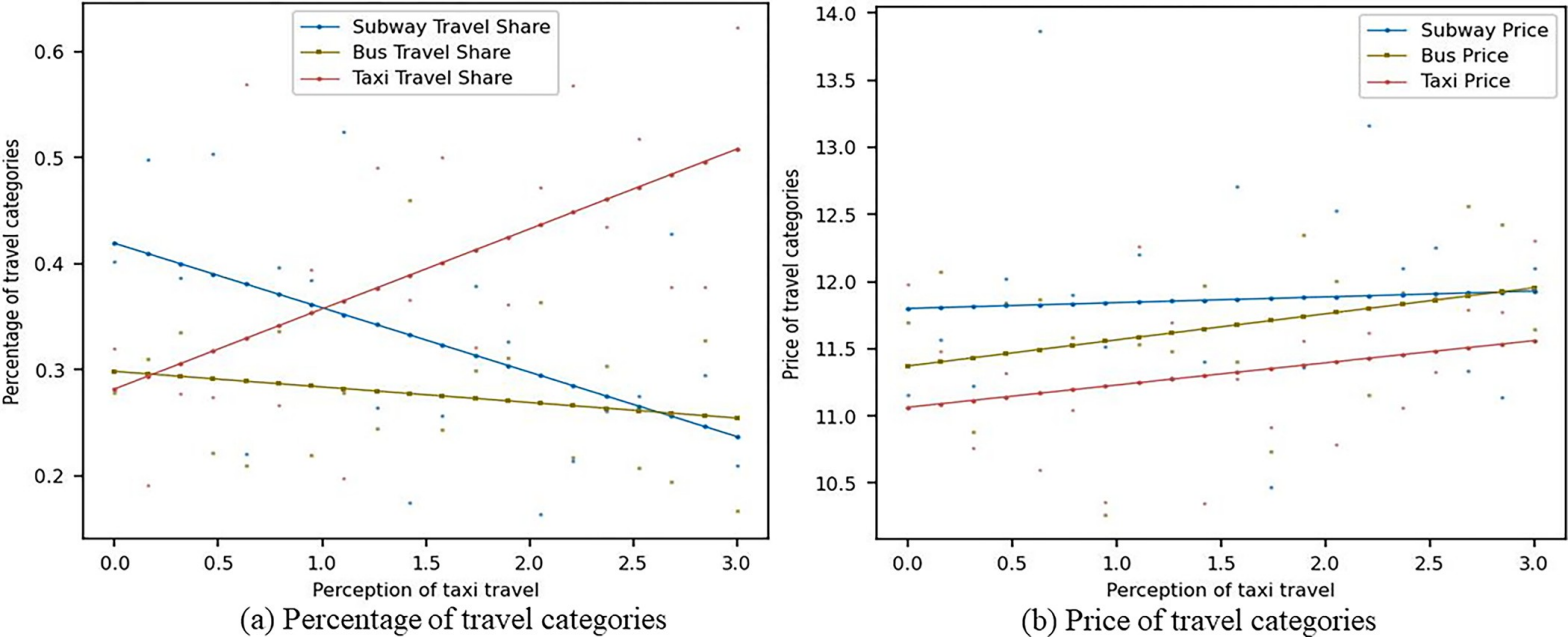
Sensitivity analysis of pa-transit travel perception with social learning.

Fig 8(A) and 8(B) shows the impact of changes in consumers’ sensitivity to pa-transit travel perception on the market shares and prices of metro, bus, and pa-transit travel modes under the scenario considering social learning. In Fig 8(A), as the sensitivity of consumers to pa-transit travel perception increases, the market share of metro travel gradually decreases from around 0.42 to 0.22, the market share of bus travel gradually decreases from around 0.3 to 0.25, and the market share of pa-transit travel gradually increases from 0.3 to around 0.55. In Fig 8(B), as the sensitivity of consumers to pa-transit travel perception increases, the prices of the metro, bus, and pa-transit all gradually increase, but they are lower than the scenario without social learning. Under the social learning scenario, the increased sensitivity of consumers to pa-transit travel perception may cause pa-transit companies to adjust their pricing strategies. Moreover, metro and bus companies may also adjust their pricing strategies to cope with market competition and changes in consumer demand. This may result in the gradual increase of prices for metros, buses, and pa-transit, but due to the presence of social learning, the overall prices are generally lower. Furthermore, under the social learning scenario, metro, bus, and pa-transit companies may focus more on improving service quality to attract consumers. This may lead to more intense competition among various travel modes, thus affecting market shares and prices.

## 5. Conclusion

In recent years, with the acceleration of urbanization and the continuous growth of transportation demand, public transportation has become increasingly important in urban travel. As the main public transportation modes, metros, buses, and pa-transit each have their own unique advantages and characteristics, together satisfying the diverse travel needs of people. However, facing fierce market competition and increasingly discerning consumers, public transportation operators need to make wise decisions in pricing strategies, service quality, and market share. Consumers’ perceptions and choice behaviors for different travel modes are of great guiding significance for operators to formulate effective strategies and for government regulatory departments to develop corresponding policies. Therefore, in-depth research on the sensitivity changes of consumers’ perceptions of metro, bus, and pa-transit travel, and their impact on market shares and prices of these travel modes, holds significant theoretical and practical value.

Therefore, we constructed a simulation analysis model for transportation operators’ pricing strategies and consumer travel choice behavior based on a multi-agent model. This model comprehensively considers the competitive relationship among metros, buses, and pa-transit, which are the three main public transportation modes, as well as consumers’ perception sensitivity and social learning behavior towards various travel modes, and employs the DDPG algorithm for simulation. Through simulation analysis and sensitivity analysis, this study explores the impact of changes in consumers’ perception sensitivity towards metro, bus, and pa-transit travel on the market shares and prices of these travel modes. The results show that as consumers’ perception sensitivity towards different travel modes increases, the market shares and prices of each mode will be adjusted accordingly. In scenarios considering consumers’ social learning behavior, the market share of metros remains at a higher level, indicating that metros have a competitive advantage in meeting consumer demand; while the market shares of buses and pa-transit are relatively lower, implying weaker positions for these two travel modes in meeting consumer demand.

In addition, this study also found that as consumers’ sensitivity to the perception of various travel modes increases, operators will invest more resources in improving service quality during the pursuit of market shares, thereby affecting the prices of each travel mode. This result has significant practical implications for public transportation operators, governments, and regulatory departments. Operators can adjust their pricing strategies and service improvement measures according to the changes in consumers’ perception sensitivity towards different travel modes, to maintain competitiveness in fierce market competition. Governments and regulatory departments can closely monitor changes in consumer behavior, develop corresponding policies and regulatory measures, and promote the healthy development of the public transportation market.

In conclusion, this study provides useful insights into consumers’ choice behavior for different travel modes and operators’ pricing strategies and offers strong support for public transportation operators, governments, and regulatory departments in adjusting pricing strategies, improving service quality, and developing relevant policies and regulatory measures amid intense market competition.

In the future, research in public transportation can explore the integration of emerging technologies and data-driven solutions to enhance efficiency, sustainability, and user experience. By harnessing advanced analytics and fostering collaboration among stakeholders, we can develop innovative strategies that respond to evolving consumer needs, promote environmentally-friendly transportation options, and ultimately contribute to the well-being of communities worldwide.

## Supporting information

S1 Table Consumer public travel questionnaire. (DOCX)

S1 Dataset (ZIP)
