# Supplementary material for: Optimal dynamic pricing for public transportation considering consumer social learning
Source: PLoS One. 2024 Jan 31;19(1):e0296263. doi: 10.1371/journal.pone.0296263 (PMC10830017; doi:10.1371/journal.pone.0296263)
Supplement: S1 Table — (DOCX) [file pone.0296263.s001.docx]

**Supporting information**

**S1. Table.** Consumer Public Travel Questionnaire

| Dear Participants,  Hello! We really appreciate your taking the time to participate in this Consumer Public Mobility Survey. This questionnaire aims to understand consumers' cognition and sensitivity to the time cost, money cost, safety, convenience, comfort and accessibility of metros, buses and pa-transit in daily travel. Based on your answers, we will analyze and evaluate the advantages and disadvantages of different means of transportation, as well as the preferences and needs of consumers when choosing travel methods. Your valuable comments will provide important support for our research and help improve public transport services and meet consumer needs.  Please rest assured that this survey is completely anonymous, and the data collected will only be used for this research and will not be disclosed to any third party. Please fill in truthfully according to your actual situation, and please try to be objective and truthful when answering.  Thanks again for your support and participation! We sincerely wish you a happy life and smooth work! | |
| --- | --- |
| 1. What kind of public transportation do you usually take? | |
| 1. Metro | |
| 1. Bus | |
| 1. Pa-transit | |
| 2. How sensitive are you to the time cost of the following means of transportation? (1 means not sensitive, 5 means very sensitive) | |
| A. Metro: 1 2 3 4 5 | |
| B. Bus: 1 2 3 4 5 | |
| C. Pa-transit: 1 2 3 4 5 | |
| 3. How sensitive are you to the money cost of the following means of transportation? (1 means not sensitive, 5 means very sensitive) | |
| A. Metro: 1 2 3 4 5 | |
| B. Bus: 1 2 3 4 5 | |
| C. Pa-transit: 1 2 3 4 5 | |
| 4. Please rate the safety, convenience, comfort and accessibility of the following means of transportation (1 means very dissatisfied, 5 means very satisfied): | |
| A. Metro： | Security: 1 2 3 4 5 |
|  | Convenience: 1 2 3 4 5 |
|  | Comfort: 1 2 3 4 5 |
|  | Accessibility: 1 2 3 4 5 |
| B. Bus： | Security: 1 2 3 4 5 |
|  | Convenience: 1 2 3 4 5 |
|  | Comfort: 1 2 3 4 5 |
|  | Accessibility: 1 2 3 4 5 |
| C. Pa-transit： | Security: 1 2 3 4 5 |
|  | Convenience: 1 2 3 4 5 |
|  | Comfort: 1 2 3 4 5 |
|  | Accessibility: 1 2 3 4 5 |
| 5. How sensitive are you to the following aspects of passenger perception? (1 means not sensitive, 5 means very sensitive) | |
| A. Security: 1 2 3 4 5 | |
| B. Convenience: 1 2 3 4 5 | |
| C. Comfort: 1 2 3 4 5 | |
| 6. How far are you from the nearest metro station, bus station and pa-transit stop? | |
| A. Metro station: ____ meters | |
| B. Bus stop: ____ meters | |
| C. Pa-transit stop: ____ meters | |
| 7. What are your average travel distances by metro, bus and pa-transit? | |
| A. Metro: ____ km | |
| B. Bus: ____ km | |
| C. Pa-transit: ____ km | |
| 8. What is your perception of the following time values? (1 means no attention, 5 means very much) | |
| A. Waiting and walking time: 1 2 3 4 5 | |
| B. Driving time: 1 2 3 4 5 | |
| 9. When you are usually waiting for the following vehicles, how long is the departure interval? | |
| A. Metro: ____ minutes | |
| B. Bus: ____ minutes | |
| C. Pa-transit: ____ minutes | |
| 10. How do you perceive the speed of the following vehicles? (1 means very slow, 5 means very fast) | |
| A. Metro: 1 2 3 4 5 | |
| B. Bus: 1 2 3 4 5 | |
| C. Pa-transit: 1 2 3 4 5 | |
| 11. What is your age range? | |
| 1. Under the age of 18 | |
| B.18-24 years old | |
| C.25-34 years old | |
| D.35-44 years old | |
| E.45-54 years old | |
| F. Over 55 years old | |
| 12. What is your occupation? | |
| 1. students | |
| B. office worker | |
| c. Freelancers | |
| D. to retire | |
| E. Others (please specify: ____) | |
| 14. What is your monthly income range? | |
| A. Below RMB 3000 | |
| B. 3000-5999 yuan | |
| C.6000-9999 yuan | |
| D.10000-14999 yuan | |
| E. More than 15,000 yuan | |
| Thank you for your participation! Your answers are very important to our research. | |
